# Tumor specific regulatory T cells in the bone marrow of breast cancer patients selectively upregulate the emigration receptor S1P1

**DOI:** 10.1007/s00262-017-1964-4

**Published:** 2017-02-21

**Authors:** Anchana Rathinasamy, Christoph Domschke, Yingzi Ge, Hans-Henning Böhm, Steffen Dettling, David Jansen, Felix Lasitschka, Ludmila Umansky, Markus H. Gräler, Jennifer Hartmann, Christel Herold-Mende, Florian Schuetz, Philipp Beckhove

**Affiliations:** 1 Division of Translational Immunology, National Center for Tumor Diseases (NCT), DKFZ, Heidelberg, Germany; 2 Department of Gynecology and Obstetrics, University Hospital Heidelberg, Heidelberg, Germany; 3 Division of Experimental Neurosurgery, Department of Neurosurgery, University Hospital Heidelberg, Heidelberg, Germany; 4 Institute of Pathology, Heidelberg University Hospital Center, Heidelberg, Germany; 5 Tissue Bank of the National Center for Tumor Diseases (NCT), Heidelberg, Germany; 6 Department of Anesthesiology and Intensive Care Medicine, Center for Sepsis Control and Care (CSCC), and Center for Molecular Biomedicine (CMB), University Hospital Jena, Jena, Germany; 7 Regensburg Center for Interventional Immunology and University Hospital Regensburg, Franz-Josef-Strauss-Allee 11, 93053 Regensburg, Germany

**Keywords:** Antigen-specific regulatory T cells, Bone marrow, Sphingosine-1-phosphate receptor 1, Exit, Breast cancer patients

## Abstract

Regulatory T cells (Treg) hamper anti-tumor T-cell responses resulting in reduced survival and failure of cancer immunotherapy. Among lymphoid organs, the bone marrow (BM) is a major site of Treg residence and recirculation. However, the process governing the emigration of Treg from BM into the circulation remains elusive. We here show that breast cancer patients harbour reduced Treg frequencies in the BM as compared to healthy individuals or the blood. This was particularly the case for tumor antigen-specific Treg which were quantified by MHCII tumor peptide loaded tetramers. We further demonstrate that decreased Treg distribution in the BM correlated with increased Treg redistribution to tumor tissue, suggesting that TCR triggering induces a translocation of Treg from the BM into tumor tissue. Sphingosine-1-phosphate receptor 1 (S1P1)—which is known to mediate exit of immune cells from lymphoid organs was selectively expressed by tumor antigen-specific BM Treg. S1P1 expression could be induced in Treg by BM-resident antigen-presenting cells (BMAPCs) in conjunction with TCR stimulation, but not by TCR stimulation or BMAPCs alone and triggered the migration of Treg but not conventional T cells (Tcon) to its ligand Sphingosine-1-phosphate (S1P). Interestingly, we detected marked S1P gradients between PB and BM in breast cancer patients but not in healthy individuals. Taken together, our data suggest a role for S1P1 in mediating the selective mobilization of tumor specific Treg from the BM of breast cancer patients and their translocation into tumor tissue.

**Electronic supplementary material:**

The online version of this article (doi:10.1007/s00262-017-1964-4) contains supplementary material, which is available to authorized users.

## Introduction

Treg are a subset of CD4+ T cells that maintain self tolerance by exerting a plethora of immune suppressive activities on various immune cells—most prominently autoreactive T effector cells. Treg also play a detrimental role in anti-tumor T-cell responses in cancer patients. Infiltration of Treg into tumors is associated with enhanced tumor growth and poor prognosis [1–3] and their therapeutic depletion through antibody-dependent cell-mediated cytotoxicity (ADCC)-proficient antibodies against a Treg-associated molecule CTLA-4 or low-dose cyclophosphamide treatment can improve anti-tumor T-cell responses and overall survival of patients [4, 5]. Conversely, in tumors promoted by chronic inflammation, Treg infiltration is beneficial as it dampens inflammation [6]. Together, data available until date clearly demonstrate the prognostic relevance of tumor infiltrating Treg [7]. This is also the case in breast cancer. Many breast cancer patients not only develop tumor antigen-specific effector T-cell responses [8–12], but also tumor specific Treg [13]. Treg accumulation in breast tumors is associated with poor overall and relapse free survival and used as an independent prognostic factor in predicting patients at high risk of relapse [14].

T-cell entry into peripheral tissues requires their exit from lymphoid organs, where they reside until their mobilization into the blood. Such an exit can be triggered by antigen-specific activation [15] or by changes in chemokine gradients and related signals [16]. The BM is an important lymphoid organ for Treg accumulation and recirculation and thus an important source of tumor infiltrating Treg [17]. However, the events triggering Treg exit from BM into PB remain largely elusive.

Here we addressed a potential role for S1P1 in regulating Treg exit from BM into PB in breast cancer patients. S1P1 mediates immune cell exit [18], particularly T cell exit from thymus and lymph nodes [19, 20]. Sawicka et al. provided evidence that treatment of mice with an S1P1 agonist resulted in an accumulation of Treg in PB and spleen but not in lymph nodes, while the same treatment resulted in increased lymph node accumulation of naïve CD4+ T cells suggesting that S1P1 could be involved in differential sequestration of Treg [21]. Cell surface residence of S1P1 is crucial to determine cell egress and is tightly regulated by S1P concentrations in the microenvironment thereby modulating trafficking patterns. It was reported that activation induces migration of lymph node Treg towards S1P [22] and that Treg tend to lose S1P1 more slowly from the surface upon activation in contrast to Tcon [23]. Hence, we here wondered whether in breast cancer patients S1P1 might be involved in the emigration of Treg populations from the BM.

Using PB and BM samples from breast cancer patients, we studied the expression, induction, and functional relevance of S1P1 and its ligand S1P in bulk and tumor antigen-specific Treg. Our findings suggest that upregulation of S1P1 on tumor antigen-specific Treg mediates their preferential exit from the BM.

## Materials and methods

### Patient samples

Collection of PB and BM samples from cancer patients and healthy donors was performed in compliance with the norms of the ethics committee [“HLA compatible allogeneic cell therapy of human tumors in mouse model’’ (approval reference number 70/99)] of the University of Heidelberg upon signed consent. Samples were withdrawn from patients (29–73-year-old Caucasians) who did not receive neo adjuvant chemotherapy and healthy donors without a history of breast cancer (21–63 years old Caucasians) after informed consent. Age range of patients and healthy donors was matched. Formalin-fixed paraffin-embedded (FFPE) tissue sections and cryosections of mammary tumors were provided by the tissue bank of the National Center for Tumor Diseases (NCT, Heidelberg, Germany) in accordance with the regulations of the tissue bank and the approval of the ethics committee of the University of Heidelberg.

### Cell isolation

PB mononuclear cells (PBMCs) and BM mononuclear cells (BMMCs) were isolated using established protocols by Ficoll (Biocoll) gradient centrifugation. Cells from the interface were collected, washed twice with X-vivo 20 medium (Lonza), and used directly for phenotyping using flow cytometry or purified and taken into culture for in vitro assays.

### Cell purification

HLADR+ primary APC were isolated using HLA-DR Microbeads Human (Miltenyi Biotec, 130-046-101) as per the manufacturer’s instructions. Alternatively, DC were differentiated in vitro as per standard protocols described earlier [24]. CD4+ Tcon and Treg were isolated using Human CD4+ CD25+ Regulatory T-cell isolation kit (Miltenyi Biotec, 130-091-301) according to the manufacturer’s instructions.

### Antibodies and flow cytometry

Tcon and Treg were phenotyped by flow cytometry (FACS Canto, BD Biosciences). Live cells were distinguished using Live/Dead Fixable Yellow Dead Cell Stain (Life Technologies, L34959) for 15 min at 4 °C followed by blocking with Kiovig (human plasma derived immunoglobulin, Baxter, PZN-4668835) at a concentration of 100 µg/ml in PBS for 15 min at room temperature. Monoclonal anti-human S1P1-APC (R&D systems, FAB2016A) and corresponding mouse IgG2B isotype control (R&D systems, IC0041A) were used at concentrations recommended by the manufacturer in PBS for 30 min at 37 °C. This was followed by surface staining for CD3-Alexa Fluor 700 (Invitrogen, CD0329), CD4-Brilliant violet 421 (BD Biosciences, 562424) and CD25-PerCp Efluor 710 (eBioscience, 46-0257-42) in PBS for 20 min at 4 °C. For some experiments, CD127-PECy7 (Biolegend, 351320) was used in conjunction to distinguish the CD127− Treg subsets. Cells were fixed and permeabilized using the fixation and permeabilization concentrate (eBioscience, 00-5123-43) and diluent (eBioscience, 00-5223-56) for 20 min at 4 °C followed by intracellular staining for FoxP3-FITC (Biolegend, 320106) in 1× permeabilization solution (eBioscience, 00-8333-56) for 20 min at 4 °C.

To detect antigen-specific T cells, DRB1*04:01 and DRB1*07:01 tetramers (PE conjugated) presenting mammaglobin as well as a negative control tetramer presenting a Class II-associated invariant chain (CLIP) peptide were obtained from NIH tetramer core facility (Emory University, Atlanta). Cells blocked with Kiovig were stained with tetramers (1 µg/100 µl PBS) for 1 h at 37 °C followed by staining with other antibodies as described above. HLA typing for the DRB1 locus was initially performed by FACS using mouse monoclonal HLADR-04 (Abcam, ab33903) or HLADR-07 (Abcam, ab34054-50) antibody for 15 min at 4 °C and detected using secondary rat anti-mouse IgM-APC (BD Pharmingen, 50676) and later reconfirmed by PCR. Data were collected using FACS Canto (BD Biosciences) with the FACS Diva software. Data analysis was done with FlowJo 8.8.6. Total number of events collected per sample varies between 0.05 mill and 0.8 mill cells. For all individual patients, Treg subsets were gated according to isotype control stainings (as shown in supplementary Fig. 1). CD25+ FoxP3+ Treg were the core population of Treg that were analyzed. In addition, CD25+ subset, CD25+ CD127− subset, and CD25+ FoxP3+ CD127− subset were also analyzed. GraphPad Prism 6 was used for graphical representations and associated statistics.

### ELISA

Plasma from PB and BM of patients were collected by centrifugation at 3000 rpm for 5 min at room temperature. The clear supernatant was used for quantifying S1P concentrations using S1P ELISA kit (Echelon Boiosciences Inc, K-1900) as per the manufacturer’s instructions.

### In vitro T-cell activation assays

T-cell activation assays were performed by coculture of purified Treg as described above with either MACS purified fresh HLADR+ primary BM APC or by differentiating DC in vitro with the standard protocols using GMCSF (560 units/ml) and IL4 (500 units/ml) for 7 days. Treg were maintained in X-vivo medium with 300 units/ml IL2 (for Tcon 100 units/ml IL2 was used) and 60 units/ml IL4. BM APC were cocultured with Treg in the ratio 1:10 along with polyclonal stimulation using the Staphylococcus aureus enterotoxin B (SEB) (1 µg/ml) or cocultured with APC alone or left unstimulated. On day 3, cells were analyzed by flow cytometry.

### Migration assay

MACS purified primary APC were cocultured with Treg/Tcon in the ratio 1:10 along with polyclonal stimulation using SEB (1 µg/ml). Unstimulated cells were used as control. On day 3 after activation, calcein staining and migration assay were performed as described by Frevert et al. [25]. Cells and chemoattractants were prepared in RPMI with 20 mM HEPES. 96 well-disposable chemotaxis plates (Neuroprobe) were coated with 10 µl of collagen (10 µg/ml in 10 mM acetic acid). Chemoattractants were added to the lower chamber, and 50,000 calcein labeled Treg were added to the upper chamber. After incubation for 1 h at 37 °C, cells that migrated to the lower well were read using a calcein reader (Perkin Elmer 2030 multi-label reader). Results obtained as fluorescence intensity units were normalized to cells that migrated to medium alone.

### Epigenetic Treg assay

Tumor areas on FFPE breast tumor sections were demarcated by Hematoxylin Eosin (H&E) stained reference slides and subsequently scraped into eppendorf tubes manually. DNA isolation and epigenetic quantification of Treg based on amplifying the Treg Specific Demethylated Region (TSDR) by qPCR were performed at Epiontis, Berlin as previously described [26]. Total Treg and CD3 T-cell numbers that infiltrated the tumor were quantified based on the number of GAPDH plasmid units obtained in each assay. Cell numbers were normalized to 1 mm^3^ tumor volume.

### Multicolour immunofluorescence staining and data acquisition using TissueFAXS

Intratumoral T-cell subpopulations were detected by a combination of primary antibodies: anti-CD3 (Dako, A0452, Host-rabbit), anti-CD8 (Clone YTC182.20, Abcam, Ab60076, Host-rat), and anti-FOXP3 (Clone 236 A/E7, Abcam, Ab20034, Host-mouse) on acetone-fixed breast tumor cryosections as described earlier [27]. Primary specific secondary antibodies (anti-rabbit Alexafluor 647, A21245; anti-rat Alexa Fluor 488, A11006; anti-mouse Alexa Fluor 555, A31570) were purchased from Life Technologies. DAPI (Invitrogen, D1306) was utilized to stain cell nuclei.

Total tissue slides were scanned on Olympus IX51 microscope equipped with a F-View II camera (both Olympus) and analyzed by the TissueQuest Cell Analysis Software package (version 4.0.1.0137, TissueGnostics GmbH). For automated analysis with TissueQuest, DAPI staining was used as a master marker for cell identification on the basis of nuclei detection. Based on H&E stained reference slides, regions of interest (ROI) were defined to distinguish between tumor and surrounding non-tumor area. All tissues were analyzed with identical parameters for detection of T cells based on nuclear size, mean staining intensity, and background threshold. Cells were visualized in scattergrams, while the cutoff between positive and negative gated cells was validated manually by backward gating on the original image.

### Statistical analyses

Distributions of data were described by mean with the standard error of the mean (SEM) or median with interquartile range as appropriate considering the form of the distribution. Consequently, the unpaired or paired *t* test or, respectively, the Wilcoxon matched pairs signed rank test were used to compare two different distributions test. Non-parametric Spearman Correlation was used to analyze the association between the frequency of Treg in BM and tumor. Two-way analysis of ANOVA with repeated measurements in both factors (concentration and stimulation status) and paired *t* tests as post hoc test was used for analyzing migration potential of Treg to S1P. *p value <0.05; **p value <0.01; ***p value <0.001; ****p value <0.0001; Differences were considered statistically significant when *p* < 0.05.

## Results

### Treg in breast cancer patients are inversely distributed between BM and tumor tissue

**Fig. 1:**
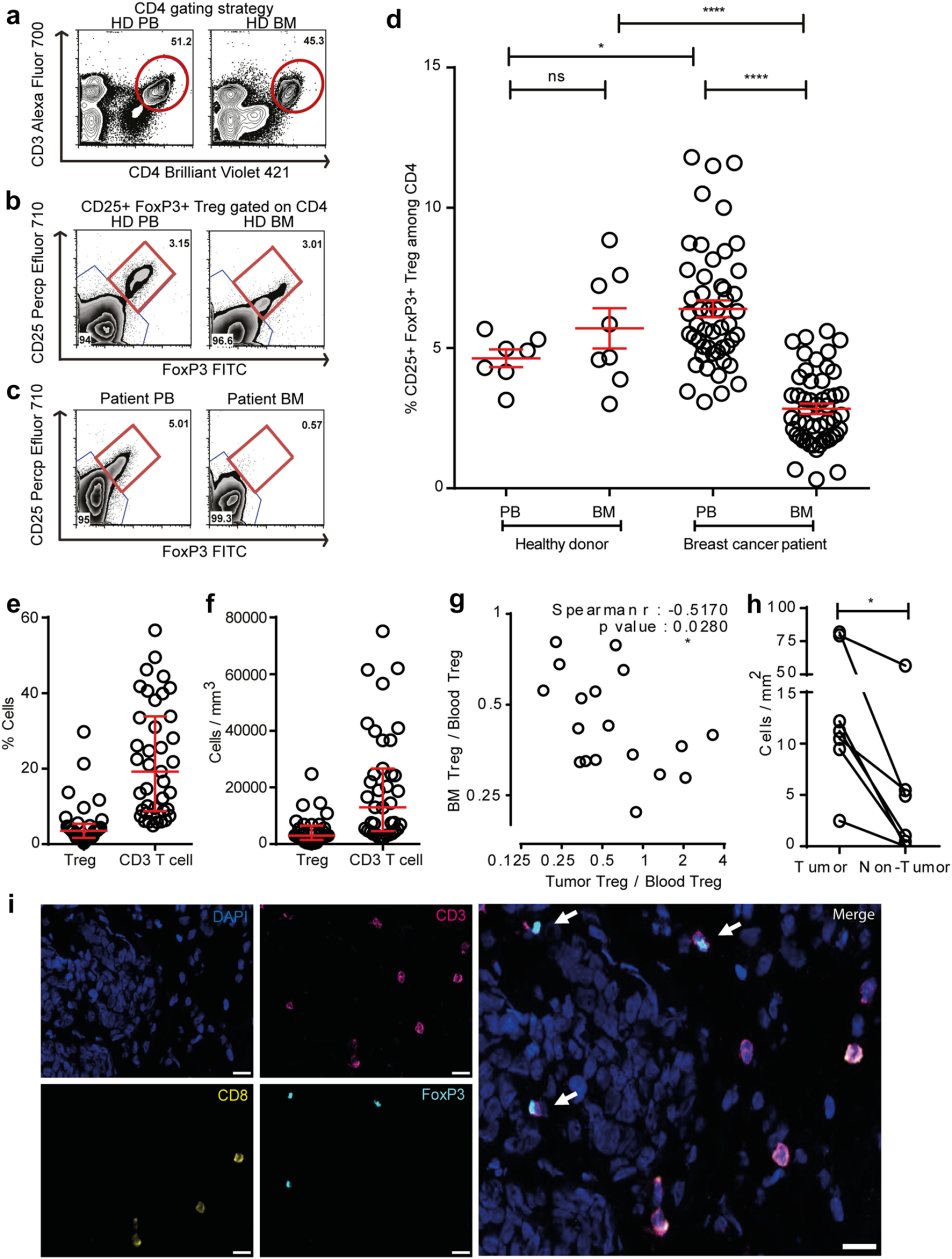
Decreased frequencies of Treg subpopulations in the BM of breast cancer patients. **a** Representative plot of a healthy donor showing the CD4+ T-cell gating strategy. **b–c** CD4+ cells were analyzed for FoxP3 and CD25 expression. CD25+ FoxP3+ cells were gated as Treg, and CD25− FoxP3− cells were gated as Tcon. Representative plots illustrating Treg frequencies in BM and PB of a healthy donor (**b**) and a breast cancer patient (**c**). *Red circle* and *square* represents the CD4 gate and CD25+ FoxP3+ Treg gate, respectively. **d** Cumulative data of CD25+ FoxP3+ Treg frequencies in PB and BM of all patients and healthy donors analyzed—Healthy donor PB (*n* = 7) and BM (*n* = 8), patients with matched PB and BM samples (*n* = 50). Data distribution in 1**d** is represented by mean with SEM. For healthy donors unpaired *t* test and for patient samples, paired *t* test was used for statistical analysis. Epigenetic PCR was performed on DNA isolated from tumor areas from FFPE sections obtained from 42 patients. Samples that passed quality control [Treg (*n* = 33) and CD3 (*n* = 40)] were taken into analysis. **e, f** Treg and CD3 T-cell percentage (**e**) and counts per mm^3^ volume of tumor (**f**). Data distribution is represented by median with interquartile range in **e** and **f. g** Graph bridging data of Treg frequencies in BM, PB, and tumor—ratio of Treg in BM to PB correlated to ratio of Treg in tumor to PB [for all patients with Treg frequencies >4.6% in PB (*n* = 18)]. Non-parametric Spearman Correlation was used for analysis (Spearman *r* = −0.5170). Axes are depicted on log 2 scale. **h**. Graph depicting Treg counts in tumor and non-tumor areas of breast tissue (*n* = 7). Lines connect data from individual patients. Non-parametric Wilcoxon matched pair signed rank test was used for statistical analysis. **i** Representative immunofluorescence staining of mammary tumor cryosection with arrows indicating CD4+ Treg and analyzed by the TissueQuest software. Cells that were DAPI+ FoxP3+ CD3+ and CD8− were defined as CD4+ Treg. Original magnification, 20×; *Scale bar* 20 µm

We first compared Treg frequencies by flow cytometry in PB and BM of healthy individuals (PB *n* = 7 and BM *n* = 8) and 50 breast cancer patients using CD25, FoxP3, and CD4 as Treg markers (Fig. 1a–c). Healthy individuals contained average Treg frequencies of 4.6% in PB, which is in accordance with published literature [28] and slightly, but not significantly higher Treg frequencies in BM (mean: 5.7%) (Fig. 1d). In breast cancer patients, we observed in accordance with published literature significantly increased frequencies of Treg in PB (mean: 6.4%) which were elevated in 82% of the cases over mean values of the healthy donor control group. Strikingly, despite their increased Treg numbers in the blood, most of the patients harboured strongly reduced Treg frequencies in the BM (mean: 2.8%), resulting in a strong change of Treg distribution between both compartments from a BM: PB ratio of 1.2 (healthy donors) to 0.4 (patients). We likewise observed lower frequencies of CD25+ among all CD4 T cells—(Supplementary Fig. 1a, g, h); and among all CD3 T cells—(Supplementary Fig. 2a–c) in the BM with a corresponding increase in the PB. Since in humans, FoxP3 can also be expressed by subsets of activated T cells, we did an additional assessment of CD127 which is expressed by Tcon but not by Treg [29, 30]. Among CD4 T cells, we also observed reduced frequencies of CD25+ FoxP3+ CD127− (Supplementary Fig. 1b–d) and CD25+ CD127− (Supplementary Fig. 1e, f) Treg in BM as compared to PB. Thus, compared to healthy donors, breast cancer patients harbor reduced frequencies of Treg in their bone marrow despite an overall increased population of Treg in the blood. This raises the question, whether in breast cancer patients—in contrast to healthy donors—Treg populations might be mobilized from the bone marrow, e.g., followed by their immigration into tumor tissue.

Treg can rapidly migrate from the PB to tumors [3, 31]. To assess whether the loss of Treg from the BM was associated with Treg accumulation in breast tumor tissue, we quantified by Epigenetic PCR Treg and CD3 T-cell infiltration in breast tumor samples of 42 patients. As shown in Fig. 1e and f, we detected a median infiltration of 3.56% Treg and 19.2% CD3 T cells among total tumor infiltrating cells resembling a calculated absolute number of 2951 Treg and 12,956 CD3+ T cells per mm^3^.

We could correlate tumor infiltrating Treg in some of these tumor samples to the frequencies of Treg in the PB and BM of the same patients. We focused on those patients who showed elevated Treg frequencies in their PB over mean values in healthy individuals (4.6%) (Fig. 1d), as in these patients, a Treg response is likely. To normalize for the overall strong inter-individual heterogeneity in absolute Treg levels among these patients, we determined for each patient the relative distribution of Treg between the BM and the tumor tissue by calculating the ratios with the respective Treg frequencies in the PB. We detected that reduced Treg frequencies in the BM significantly correlated with increased Treg infiltration in the corresponding breast tumors (Fig. 1g-i), suggesting that in breast cancer patients, Treg populations might shift from one to the other compartment. Such Treg accumulation in tumors appears to be tumor tissue selective as we detected by microscopic analysis only little Treg infiltration in normal breast tissue of the same patients (Fig. 1h).

### Tumor antigen-specific Treg in the BM express high levels of S1P1

**Fig. 2:**
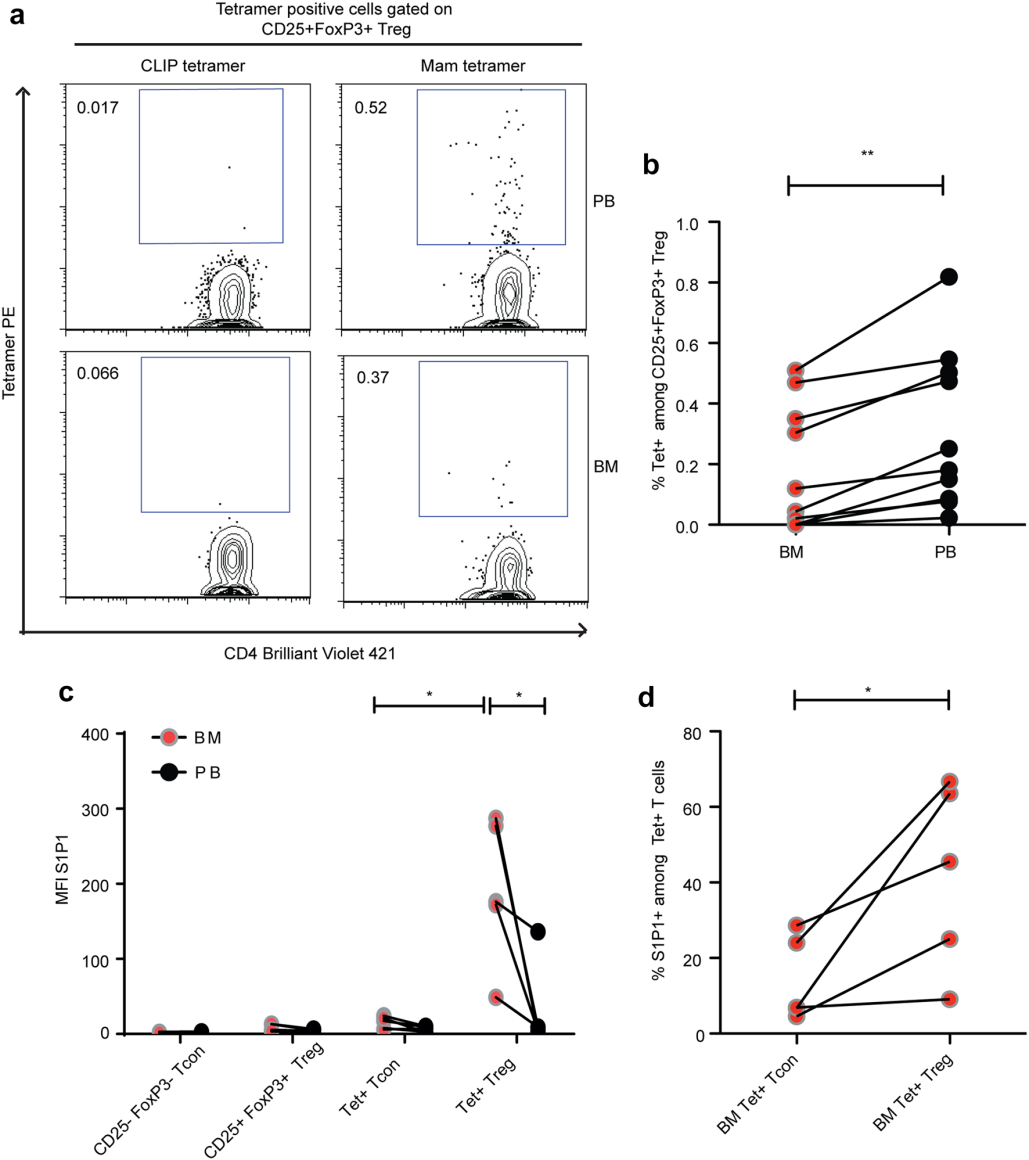
Mammaglobin specific Treg in BM expresses S1P1. **a** Dot plot depicting frequencies of mammaglobin specific Treg in PB and BM of a breast cancer patient. **b** Cumulative data of mammaglobin specific Treg frequencies in PB and BM of breast cancer patients (*n* = 10). **c** *Graph* showing MFI of S1P1 expression on bulk and antigen-specific Tcon and Treg in PB and BM and **d** Graph showing percentage of S1P1 positive BM Tcon and Treg from all DR04 and DR07 patients analyzed (*n* = 5). Data distribution is represented by mean with SEM in **b–d**. Paired *t* test was used for statistical analysis in **b–d**.* Lines* connect data from individual patients

One major trigger of T-cell emigration from lymphoid organs is antigen-specific stimulation [15, 32]. We thus reasoned that the population of tumor antigen-specific Treg would be under-represented among BM-resident Treg in case T-cell receptor stimulation also triggered the observed selective reduction of Treg in the BM. To detect and phenotype breast tumor specific CD4+ Treg and Tcon cells by flow cytometry, we had previously developed MHC II tetramers (HLADR 04:01 and HLADR 07:01) loaded with MHC-allele restricted peptides derived from the breast tumor associated antigen mammaglobin I [13]. With these MHC II tetramers, we successfully detected increased frequencies of tumor specific Treg in the blood of breast cancer patients, while the occurrence of tumor antigen-specific Treg in healthy individuals’ blood is rare (0.07%) [13]. In accordance with our previous findings, we detected populations of tumor antigen-specific Treg in the PB of breast cancer patients. We also detected tumor specific Treg in their BM, but at significantly lower frequencies than in the PB (Fig. 2a, b). Thus, tumor specific Treg were reduced among BM-resident Treg populations, suggesting that the loss of Treg from the BM of breast cancer patients involves, at least partially, TCR-mediated signals. To understand a possible mechanism that could mediate the mobilization of Treg from BM, we studied the expression of S1P1 on Treg (S1P1 antibody staining was tested on HTC Rat hepatoma cell line over expressing human S1P1 prior to staining on antigen-specific Treg in BM and PB—Supplementary Fig. 3). Interestingly, we observed a high expression of S1P1 only on tumor antigen-specific Treg in the BM, while S1P1 expression was low on CD25+ FoxP3+ Treg, CD25-FoxP3-Tcon, tumor specific Tcon in PB and BM, and tumor specific Treg in PB (Fig. 2c, d). Thus, in breast cancer patients, S1P1 expression characterizes a population of BM-resident tumor antigen-specific Treg, suggesting that S1P1 expression is related to TCR stimulation of Treg in the BM.

### TCR stimulation induces S1P1 expression on BM Treg

**Fig. 3:**
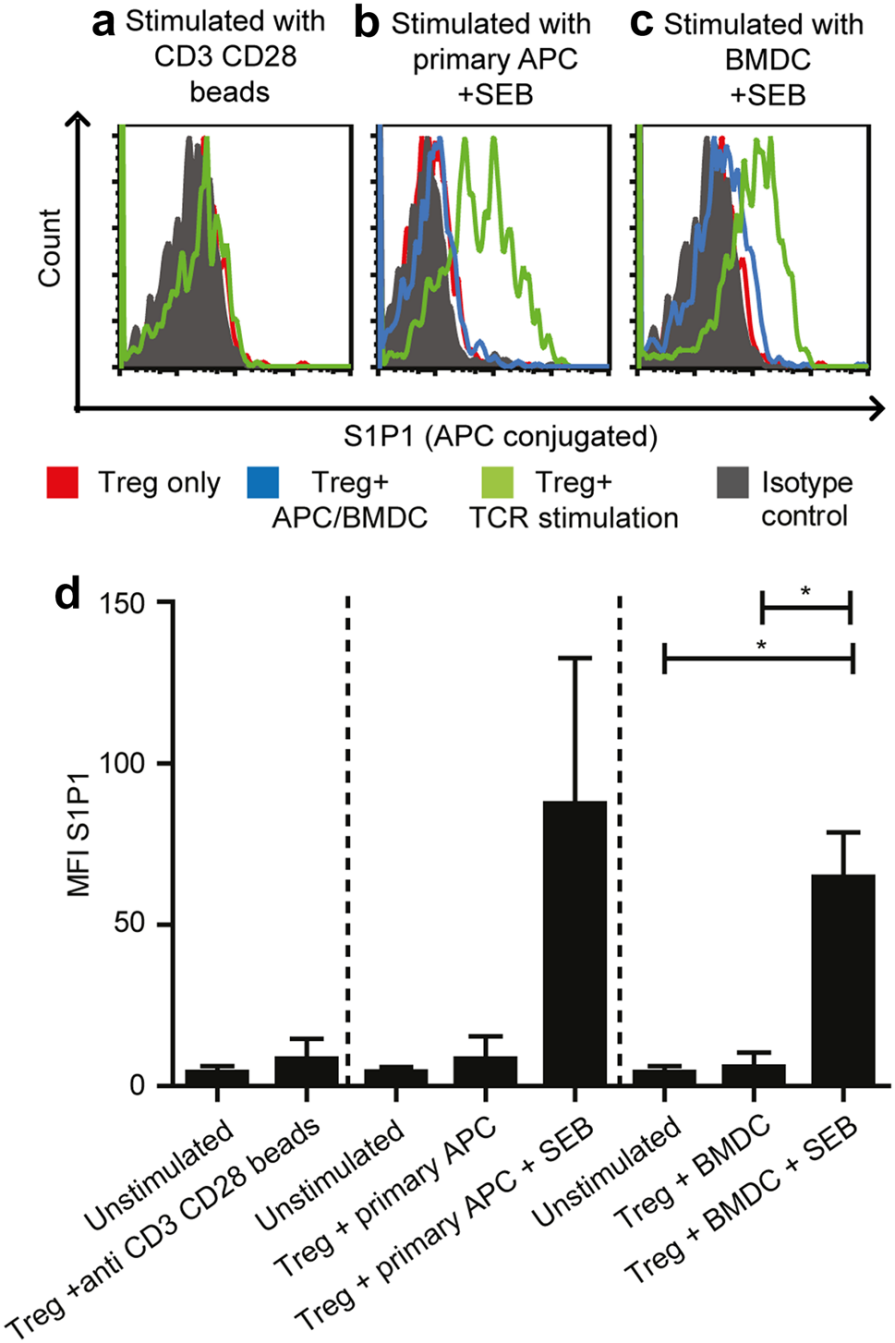
Antigen-specific TCR stimulation induces S1P1 expression on BM Treg. **a** Histogram of S1P1 expression on a patient’s BM Treg stimulated with anti-CD3 anti-CD28 beads (*n* = 3). **b** Histogram depicting S1P1 expression on a patient’s BM Treg stimulated with purified primary APC alone (*n* = 2) or primary APC with SEB (*n* = 5). **c** Histogram depicting S1P1 expression on a patient’s BM Treg stimulated with in vitro differentiated BMDC alone or BMDC with SEB (*n* = 4) three days after activation. *Red line* in the histograms depict S1P1 expression on unstimulated Treg alone. *Green line* in the histograms refer to S1P1 expression on TCR stimulated Treg. *Blue line* (only in **b** and **c**) refers to S1P1 expression on Treg that were cocultured with APC or BMDC alone without the polyclonal SEB stimulus. *Solid grey histogram* represents S1P1 isotype control. **d** Cumulative data from three different stimulation settings. Paired *t* test was used (when *n* is > or =4) for statistical analysis

As S1P1 was selectively upregulated on antigen-specific Treg in the BM, we studied the impact of TCR stimulation on S1P1 expression. To this end, we purified Treg from the BM and polyclonally stimulated their TCRs using anti-CD3 and anti-CD28-coated beads (Fig. 3a). We also assessed the impact of antigen-presenting cell populations in the BM during polyclonal TCR stimulation using SEB for cross-linking the TCRs to HLA-DR molecules [33] presented by either purified BM-derived autologous HLA-DR+ primary APC (Fig. 3b) or in vitro generated BM-derived dendritic cells (BMDC) (Fig. 3c). We assessed S1P1 expression 3 days after the respective stimulation procedure as Pham et al. reported that stimulated murine effector T cells expressed the highest levels of S1P1 after 3 days of activation [16]. As shown in Fig. 3a–d, TCR stimulation using anti-CD3 anti-CD28-coated beads alone or coculture with either BM-derived primary APC or in vitro differentiated BMDC alone did not induce S1P1 expression on Treg. However, TCR stimulation by BM-derived APC populations that presented the bacterial superantigen SEB resulted in strong S1P1 expression. These results demonstrate that on the one hand side, antigen-specific stimulation through the TCR is crucial for upregulating S1P1 expression on Treg and on the other hand that the presence of APC together with TCR stimulation is necessary for S1P1 upregulation on Treg.

### Activated Treg migrate to S1P in vitro

**Fig. 4:**
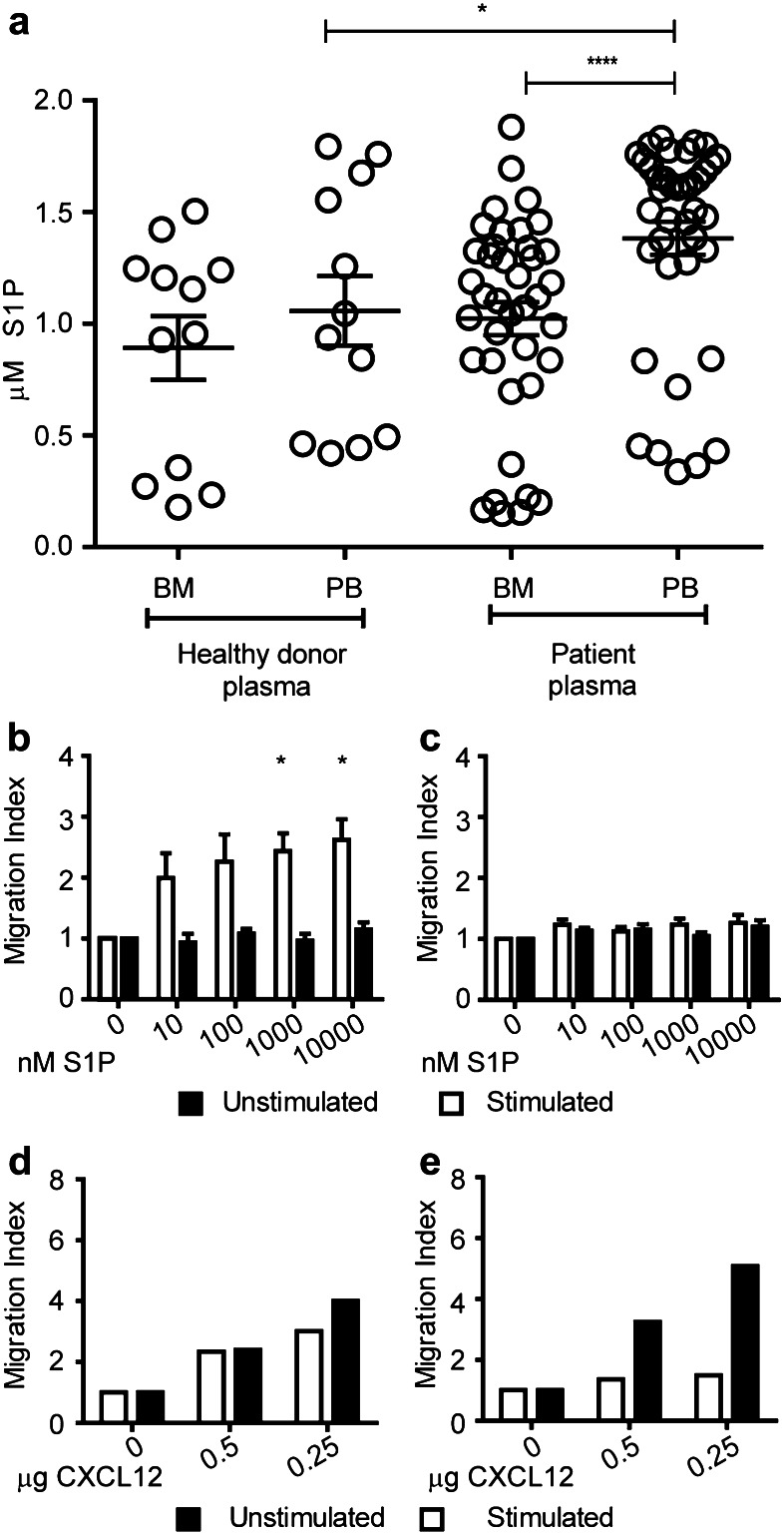
In vitro activated BM Treg migrate to S1P. **a** Quantification of S1P concentrations in PB and BM (*n* = 12) plasma of healthy donors and breast cancer patients (*n* = 39) by ELISA. For healthy donors unpaired *t* test and for patient samples, paired *t* test was used for statistical analysis. **b–c** Migration index (flourescence intensity units of cells that migrated to chemoattractant/flourescence intensity units of cells that migrated to medium alone) of BM Treg (**b**) and Tcon (**c**) towards S1P (*n* = 4). **d–e** Migration index of Treg (**d**) and Tcon (**e**) towards CXCL12 as control (*n* = 2). Distribution of data is represented by mean with SEM. Two-way analysis of ANOVA with repeated measurements was performed with concentration as one factor and stimulation status as the other factor. Concentration (*p* value 0.0010), stimulation (*p* value 0.0494), and interaction between concentration and stimulation (*p* value 0.0155) suggesting a different influence of stimulation depending on the concentration. Post hoc paired *t* tests showed a significant influence of stimulation in the two highest concentrations (1000 and 10,000 nM S1P)

We next assessed the concentrations of the ligand S1P in the BM and PB [34] of healthy donors and breast cancer patients. PB plasma S1P levels were significantly elevated in breast cancer patients compared to healthy donors. In contrast, S1P concentration in BM was lower than in PB of patients but similar to S1P concentrations present in BM of healthy individuals. Nevertheless, the highly elevated S1P concentration in patients’ PB resulted in a pronounced S1P concentration difference between the BM and the PB in breast cancer patients of 400 nM (Fig. 4a). We, therefore, assessed whether BM-derived Treg could migrate towards S1P concentrations similar to physiological levels of S1P present in breast cancer patients. To this end, we purified Treg and Tcon from the BM of breast cancer patients and tested their migration towards different S1P concentrations. To assess a potential impact of TCR stimulation on the migration towards S1P, we subjected aliquots of the T cells to polyclonal TCR stimulation by coculturing them with purified autologous primary APC from the BM which were loaded with SEB.

We observed enhanced migration to increasing concentrations of S1P only in TCR stimulated Treg (Fig. 4b). In contrast, Tcon did not migrate to S1P irrespective of their activation status (Fig. 4c). Unstimulated as well as stimulated Treg migrated well towards CXCL12 used as a positive control in the migration assays confirming that unstimulated Treg were generally capable of responding to chemotactic stimuli (Fig. 4d). Tcon migrated to CXCL12 in unstimulated conditions but upon activation, the migration to CXCL12 was very much reduced (Fig. 4e). This was a reflection of the downregulation of the CXCL12 receptor CXCR4 upon activation (data not shown). Cumulatively, these results demonstrate that activated BM Treg inherently sense and migrate towards S1P.

## Discussion

While increased infiltration of Treg into solid tumors has often been described, factors driving their migration from lymphoid organs into cancerous tissues largely remain elusive. Previous work published by Peters et al. [35] suggested that Treg trafficking patterns might be strongly perturbed in solid tumor patients. A potential role of the BM for Treg recirculation in cancer patients has been already suggested by Zhao et al. [36]. Here, we show for the first time in breast cancer patients, major changes in Treg distribution between BM, PB, and tumor. This was characterized by an overall increase in PB, markedly decreased Treg frequencies in BM and an associated Treg accumulation in tumor tissue, suggesting that Treg might be mobilized from the BM into the blood and tumor. Because of high variations in overall Treg abundance between the breast cancer patients which might reflect different dynamics in Treg generation and because of potentially very short recirculation periods of activated, mobilized Treg in the blood, we could not directly identify an inverse correlation between their frequencies in the blood and bone marrow. However, we could show that in breast cancer patients, Treg distribution is inversely correlated between bone marrow and tumor tissue.

An exploratory assessment of CD25+ FoxP3+ Treg in 50 patients revealed that this characteristic Treg distribution pattern was observed in patients with different grades (Grades I, II, and III) with no significant differences with varying disease grades (data not shown). Interestingly, tumor antigen-specific Treg were significantly under-represented in the BM compared to the blood—pointing to the possibility of a link between TCR stimulation and Treg depletion in the BM. This was corroborated by our finding of selective S1P1 expression on tumor antigen-specific BM Treg. However, our in vitro activation experiments showed that TCR stimulation by CD3 CD28-coated beads alone did not induce S1P1 expression in Treg but that S1P1 surface expression on Treg required additional signals provided by APC emphasizing their importance in regulating S1P1 expression. In this regard, Shannon et al. have attributed a role for CCL19 in inducing S1P1 expression on murine T lymphocytes [37]. As CCL19 can be secreted by APC subsets [38], it would be interesting to unravel the nature of putative CCL19+ BM APC that could potentially present breast tumor antigens to Treg.

Since extracellular S1P is required for signaling via S1P1 on the cell surface, the exploitation of such S1P1 mediated mobilization of antigen specific Treg seems to be based on two conditions; first -a Treg selective induction of S1P1 through the presentation of cognate antigen by APC in the BM, and second—increased concentrations of S1P in PB plasma that have been previously reported in patients with solid tumors [39]. Here, we show that in breast cancer patients, BM S1P levels are not elevated despite increased PB plasma S1P levels creating an S1P gradient between the BM and PB. Multiple cell types that can secrete S1P [40] and produce sphingosine kinases that regulate the phosphorylation and release of S1P [41] have been described and might contribute to the observed S1P gradient between BM and PB in cancer patients.

A putative role for S1P1 in regulating Treg trafficking from human BM to PB has not been studied so far, although S1P1 surface expression has been reported on human CD4+ T cells subjected to serum starvation [42]. In our in vitro migration experiments, only TCR stimulated Treg migrated to S1P, whereas resting Treg did not. This result is in accordance with the work of Ishimaru et al. who previously reported that murine lymph node Treg activated with anti CD3 antibody migrated to S1P [22], thus emphasizing the importance of Treg activation to respond to S1P signals.

Our finding that S1P1 was scarcely expressed by tumor antigen-specific Tcon but strongly and specifically induced in tumor antigen-specific Treg hints towards a role for BM APC in the regulation and maintenance of peripheral tolerance—e.g., through mobilizing populations of self reactive Treg for dampening chronic inflammatory processes at peripheral sites. The reduced migratory potential of activated Tcon to S1P could be explained by work of Liu et al. who demonstrated that after TCR stimulation, Treg maintain S1P1 surface expression, while Tcon rapidly loose S1P1 expression [23]. Taken together, our study suggests that BM may be an important site for the activation of tumor antigen-specific Treg that get preferentially mobilized from the BM into PB via S1P1. Increased PB plasma S1P levels as well as the capacity of APC to induce S1P1 expression on Treg through TCR stimulation may contribute to such peripheral tolerance mechanism. In cancer patients, this preferential mobilization of antigen-specific Treg mediated by S1P1 from the BM might act as a preceding step in the Treg trafficking process resulting in their accumulation in tumor thus contributing to poor clinical responses. Therefore, disrupting increased S1P levels prevailing in PB plasma of cancer patients may represent a future therapeutic strategy to contain Treg within the BM thereby reducing Treg infiltration into tumors.

### Electronic supplementary material

Below is the link to the electronic supplementary material.

Supplementary material 1 (PDF 901 KB)

## Abbreviations

ADCC: Antibody-dependent cell-mediated cytotoxicity
APC: Antigen-presenting cells
BM: Bone marrow
BMAPCs: BM-resident antigen-presenting cells
BMMCs: Bone-marrow mononuclear cells
CLIP: Class II-associated invariant chain peptide
FFPE: Formalin-fixed paraffin embedded
HD: Healthy donor
Mam: Mammaglobin
PB: Peripheral blood
PBMCs: Peripheral blood mononuclear cells
S1P: Sphingosine-1-phosphate
S1P1: Sphingosine-1-phosphate receptor 1
SEB: Staphylococcus aureus enterotoxin B
Tcon: Conventional CD4+ T cells
Tet: Tetramer
Treg: Regulatory T cell(s)
TSDR: Treg specific demethylated region
