# Supplementary material for: Tumor specific regulatory T cells in the bone marrow of breast cancer patients selectively upregulate the emigration receptor S1P1
Source: Cancer Immunol Immunother. 2017 Feb 21;66(5):593–603. doi: 10.1007/s00262-017-1964-4 (PMC5406429; doi:10.1007/s00262-017-1964-4)
Supplement: Supplementary file 1 — Supplementary material 1 (PDF 901 KB) [file 262_2017_1964_MOESM1_ESM.pdf]

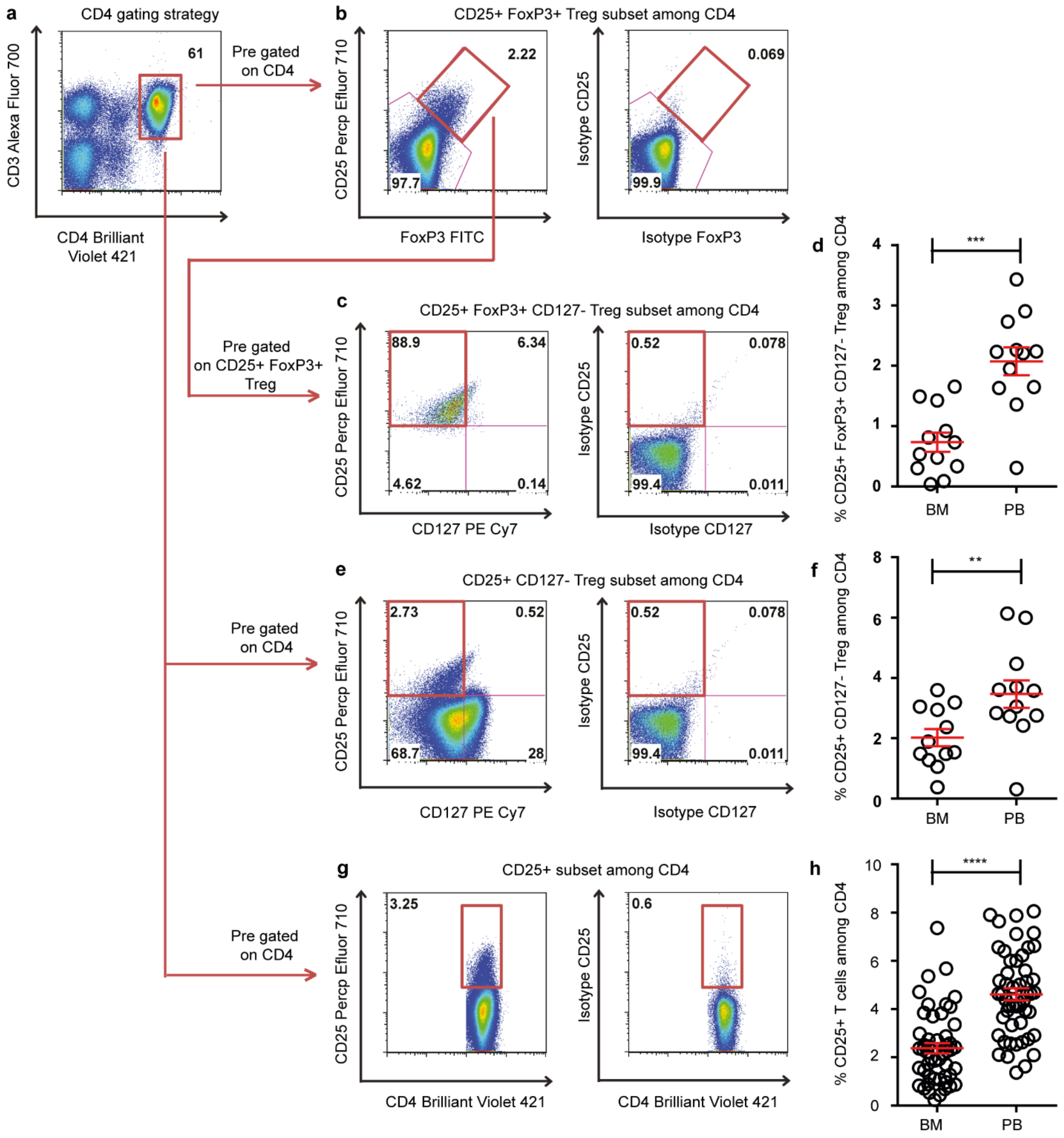

Supplementary Figure 1. Decreased frequencies of Treg subpopulations in the BM of breast cancer patients.

a. Representative Dot plots showing CD4 gating strategy. CD4+ cells were analyzed for different subsets of Treg. **b-d**. Gate (depicted in red) in (**b**) represents CD25+FoxP3+ Treg and (**c**) represents CD127- subset among CD25+FoxP3+ Treg. Graph (**d**) represents corresponding frequencies of CD25+FoxP3+CD127- Treg (among all CD4). **e-f**. Representative plot showing CD25+CD127- Treg among all CD4 (**e**) and graph showing frequencies of CD25+CD127- Treg in BM and PB among all CD4 T lymphocytes (**f**). **g-h**. Representative plot showing CD25+ T cells among all CD4 (**g**) and graph showing frequencies of CD25+ T cells in BM and PB (**h**). Panels **b**, **c**, **e**, **g** also encompass isotype control stainings that were used to set the gates for the different Treg subsets. Red squares indicate the gated population of interest. Data distribution in all graphs is represented by mean with SEM and paired t-test was used for statistical analysis.

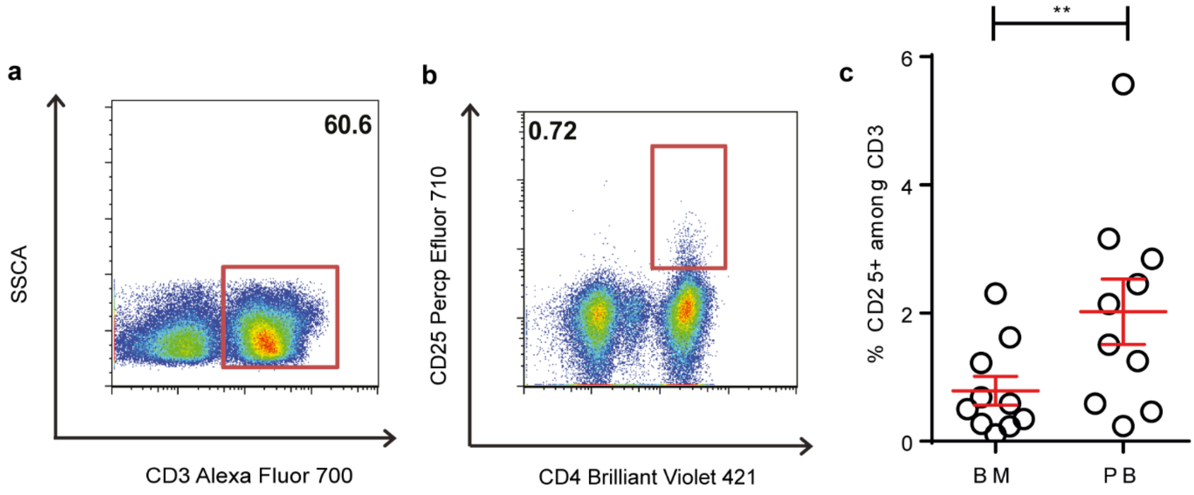

Supplementary Figure 2. CD4+CD25+ subset analysis in PB and BM of breast cancer patients

a-c. Representative Dot plots showing CD3 T cells (**a**) further analyzed for CD4 and CD25 expression (**b**). The red gate in (**a**) shows CD3 T cells and the gate in (**b**) shows CD25+CD4+ subset. Frequencies of CD25+CD4+ subset among all CD3 are depicted in the graph (**c**). Data distribution is represented by mean with SEM and paired t-test was used for statistical analysis.

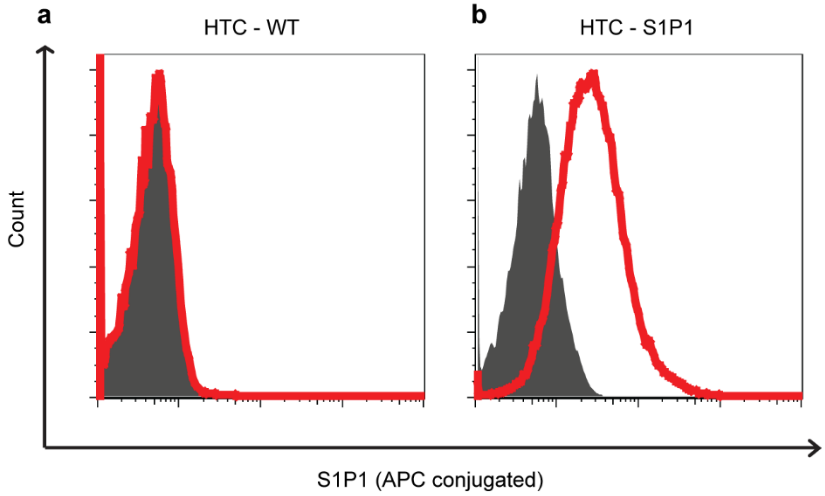

Testing S1P1 antibody on S1P1 over expressing cell line:

Staining of S1P1 antibody was performed at 37°C for 30 minutes on wild type HTC Rat hepatoma cells (HTC-WT) (**a**) and HTC cells that were stably transduced with human S1P1 (HTC-S1P1) (**b**). S1P1 staining was clearly observed on HTC cells stably transduced with human S1P1.
